# Comparison of a drug-eluting balloon first and then bare metal stent with a drug-eluting stent for treatment of *de novo* lesions: study protocol of a randomized controlled trial

**DOI:** 10.1186/1745-6215-14-38

**Published:** 2013-02-08

**Authors:** Sang-Don Park, Chang-Hwan Yoon, Il-Young Oh, Jung-Won Suh, Young-Suk Cho, Tae-Jin Youn, Dong-Ju Choi, In-Ho Chae

**Affiliations:** 1 Division of Cardiology, Department of Internal Medicine, Seoul National University Bundang Hospital, 82 Gumi-ro, 173 Bein-gil, Bundang-gu, Seongnam-si, Gyeonggi-do, 463-707, Republic of Korea

**Keywords:** Drug-eluting balloon, Bare metal stent, Drug-eluting stent, In-segment late loss

## Abstract

**Background:**

The use of a drug-eluting balloon for the treatment of *de novo* coronary artery lesions remains to be evaluated. A previous trial in patients with stable and unstable angina comparing a bare metal stent mounted on a drug-eluting balloon with a sirolimus-eluting stent failed to meet the prespecified non-inferiority criteria versus the sirolimus-eluting stent. The stent struts of a bare metal stent pre-mounted on a drug-eluting balloon may prevent the appropriate delivery of drugs to the vessel wall and may result in reduced efficacy. In the present study we will therefore evaluate the efficacy of a drug-eluting balloon for treating *de novo* coronary artery lesions using a strategy designed to uniformly deliver drug to the vessel with a bare metal stent.

**Methods/Design:**

The Comparison of Drug-Eluting Balloon first study is a prospective, randomized, open-label trial designed to demonstrate the non-inferiority of first using a drug-eluting balloon (Sequent^®^ please; B. Braun, Melsungen, Germany) followed by a bare metal stent (Coroflex^®^ Blue; B. Braun) compared with using a drug-eluting stent (Resolute Integrity™; Boston Scientific, Natick, MA, USA) for *de novo* coronary artery lesions. The primary endpoint of the study is in-segment late loss at 9 months measured by quantitative coronary angiography. Secondary endpoints include angiographic findings such as angiographic success, device success, binary angiographic restenosis, and clinical outcomes such as procedural success, all-cause death, myocardial infarction, target vessel revascularization, target lesion revascularization, and stent thrombosis. A total of 180 patients will be enrolled in the study.

**Discussion:**

The Comparison of Drug-Eluting Balloon first study will evaluate the clinical efficacy, angiographic outcomes and safety of a drug-eluting balloon first followed by a bare metal stent compared with a drug-eluting stent for the treatment of *de novo* coronary artery lesions.

**Trial registration:**

Clinical Trials.gov: NCT01539603

## Background

Since percutaneous coronary intervention (PCI), using balloon angioplasty and bare metal stents (BMS), was introduced, restenosis of a lesion treated percutaneously remains a matter of concern, especially when BMS are used [1,2]. Although the use of drug-eluting stents (DES) has reduced the occurrence of restenosis and subsequent need for repeat revascularization [3], the incidence of stent thrombosis with DES appeared to be high due to incomplete re-endothelialization and ongoing vascular inflammation after DES implantation [4,5]. Persistent polymer in the vessel wall may aggravate pathogenesis of atherosclerosis for an extended period of time [6]. In addition, a late catch-up phenomenon or accelerated neoatherosclerosis over time in first-generation DES have raised concerns about the extensive use of DES [7,8]. These types of disappointing results with DES may be explained by the fact that an optimal concentration of the drug is not uniformly reached at the vessel wall [9], because the DES contains a relatively low dose of the drug that is slowly released over an extended period of time from the polymer stent coating.

The drug-eluting balloon (DEB), a nonstent-based local antiproliferative drug-delivery system, has been developed recently to overcome the limitation of DES; that is, release of eluting drugs based on a stent strut. In contrast to DES, this DEB works by locally releasing a controlled dose of drug that is homogeneously distributed to the entire injured vessel wall and not limited to the surface area adjacent to a stent strut [10]. Preclinical studies of the DEB have demonstrated a significant reduction in neointimal formation, as compared with DES [11,12]. Compared with a standard uncoated balloon, a paclitaxel-coated balloon significantly reduced neointimal proliferation and the need for target vessel revascularization in an in-stent restenosis setting [9]. Furthermore, the DEB was superior to DES with late lumen loss and was associated with fewer adverse clinical events in treatment of coronary in-stent restenosis [13].

Among the DEB studies, Paclitaxel Eluting PTCA Balloon in Coronary Artery Disease (PEPCAD) I was the first trial using a DEB in *de novo* coronary lesions in a high-risk patient population. The result of this trial encouraged a randomized clinical trial comparing the DEB in small coronary vessels and bifurcation lesions. Promising clinical data are available for the stand-alone use of the DEB in small vessel coronary disease [14] and bifurcation lesions [15]. In *de novo* coronary lesions, the PEPCAD III trial was the first to compare DEBs and pre-mounted BMS in combination with a sirolimus-eluting stent in patients with stable and unstable angina. However, per-protocol analysis of this trial revealed that the strategy of BMS pre-mounted on DEBs did not meet the non-inferiority criteria versus the sirolimus-eluting stent [16]. In the PEPCAD III trial, since BMS pre-mounted on DEBs were implanted in the *de novo* coronary lesion, drugs may be inappropriately delivered and unevenly distributed to the diseased vessel wall due to the pre-mounted stent strut. This inconsistency might diminish the efficacy of the DEB that had been shown in the previous studies.

We therefore designed a clinical study with a different protocol in which first the DEB is deployed followed by BMS implantation in comparison with DES implantation alone. Using this protocol in the treatment of *de novo* coronary lesions, we expect to demonstrate the combined efficacy of DEBs and BMS in the treatment of *de novo* coronary lesions.

### Study objectives

The primary objective of the DEB first study is to evaluate clinical efficacy, angiographic outcomes and safety of the DEB first followed by BMS implantation compared with a drug-eluting stent for treatment of *de novo* coronary lesions.

## Methods

### Study design

This trial will be a prospective, randomized, open-label trial to demonstrate the non-inferiority of first using a paclitaxel-coated balloon (Sequent^®^ please; B. Braun, Melsungen, Germany) followed by BMS implantation (Coroflex^®^ Blue; B. Braun) compared with a zotarolimus-eluting stent (Resolute Integrity™; Boston Scientific, Natick, MA, USA) in *de novo* coronary lesions.

**Figure 1:**
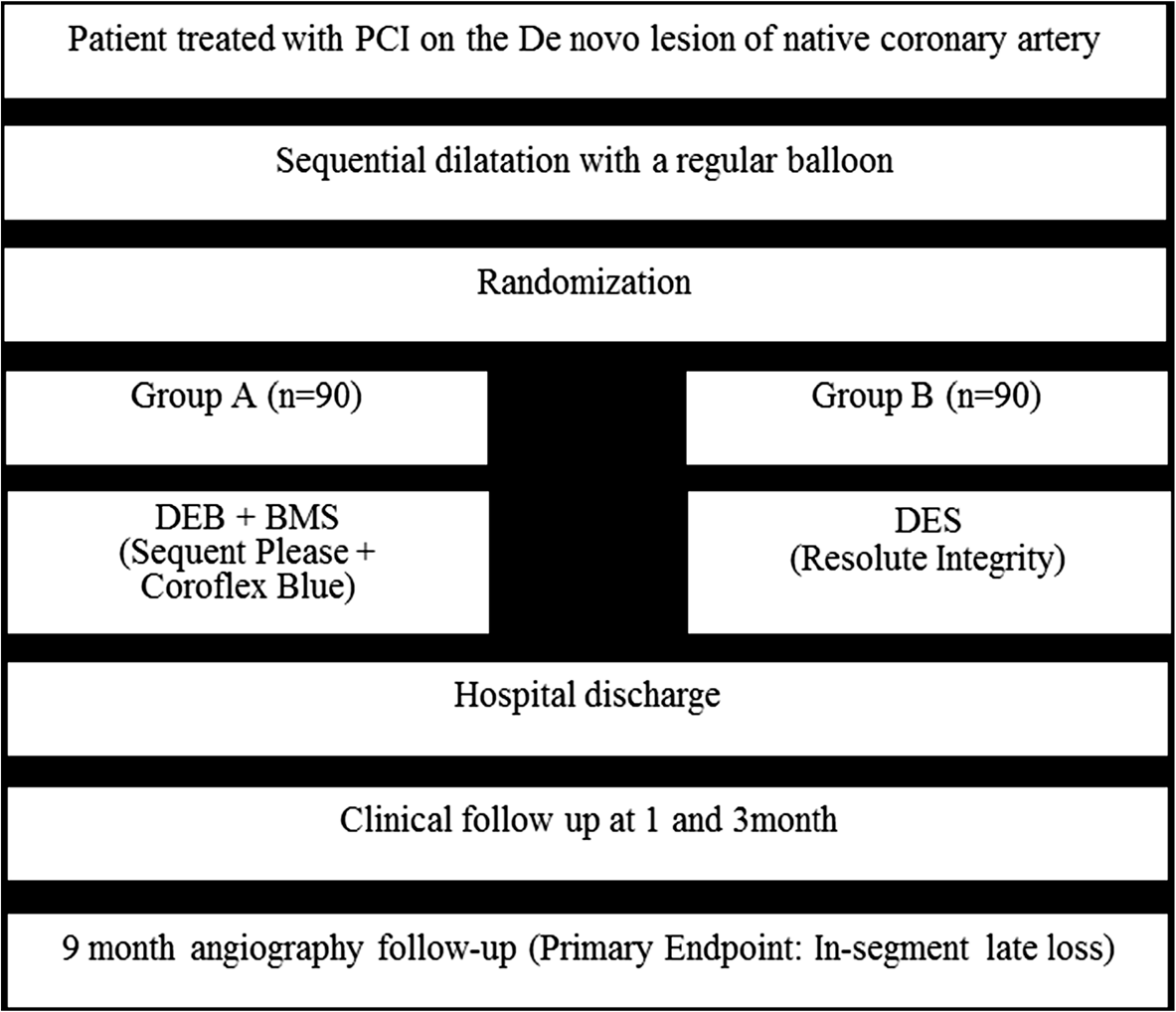
**Drug-eluting balloon first study algorithm.** BMS, bare metal stent; DEB, drug-eluting balloon; DES, drug-eluting stent; PCI, percutaneous coronary intervention.

The protocol of the trial has been registered online (NCT01539603) [17] and a brief flowchart of the entire study is summarized in Figure 1. The schedule of events for this trial is described in Table 1.

**Table 1:** Schedule of events

	**Baseline**	**Post-procedure**	**Follow-up**			
			**30 days± 2 weeks**	**3 ± 1 months**	**9 ± 2 months**	**12 ± 2 months**
Medical/clinical history (age, sex, risk factors, clinical diagnosis, angina status, cardiac history)	**X**				**X**	
Informed consent^a^	**X**					
Inclusion/exclusion criteria	**X**					
Brief physical examination	**X**				**X**	
Vital status	**X**		**X**		**X**	**X**
Weight, height	**X**					
Twelve-lead ECG^b^	**X**					
Angiogram	**X**				**X**^c^	
Complete blood count	**X**		**X**		**X**	**X**
Electrolytes, LFT	**X**		**X**		**X**	**X**
Creatinine, BUN	**X**		**X**		**X**	**X**
hs-CRP	**X**		**X**		**X**	**X**
Fasting plasma triglycerides, HDL, total cholesterol	**X**		**X**		**X**	**X**
Fasting glucose level^d^	**X**		**X**		**X**	**X**
HgbA1C^e^	**X**		**X**		**X**	**X**
Pregnancy test (if applicable)	**X**					
Medications	**X**		**X**	**X**	**X**	**X**
CK, CK-MB, Troponin I^f^	**X**	**X**				
proBNP	**X**		**X**			**X**

BUN, blood urea nitrogen; CK, creatine kinase; LFT, liver function test; HDL, high-density lipoprotein; Hgb, hemoglobin; hs-CRP, high-sensitivity C-reactive protein; proBNP, pro-brain natriuretic peptide. ^a^The informed consent may be signed either prior to the diagnostic angiogram or after the diagnostic angiogram. ^b^Additional electrocardiograms (ECGs) will be performed at 60 ± 30 minutes post procedure. ECG at follow-up visits will only be obtained when clinically indicated, such as recurrent chest pain, ischemia, or significant arrhythmias, heart failure or other signs or symptoms of clinical instability. ^c^Routine follow-up angiography will be recommended at 9 months, but it can be performed at 9 ± 2 --> 9 ± 3 months. Unscheduled angiograms ≥6 months after the index procedure will be considered as the 9-month follow-up angiogram in final analysis. ^d^Measurement may be made later, before discharge, when the patient is in a fasting state. ^e^For patients with diagnosed diabetes mellitus. ^f^Cardiac enzymes should be followed up for at least 24 hours in patients indicated clinically, such as recurrent chest pain, ischemia, or significant arrhythmias, heart failure or other signs or symptoms of clinical instability. Otherwise the decision is up to the operator. If follow-up is made, enzymes must be followed every 8 hours for at least 24 hours post index procedure.

### Endpoints

The primary endpoint of the study is in-segment late loss at 9 months measured by quantitative coronary angiography. Secondary endpoints include angiographic findings such as angiographic success, device success and binary angiographic restenosis and clinical outcomes such as procedural success, all-cause death, myocardial infarction, target vessel revascularization, target lesion revascularization and stent thrombosis.

### Patient population

Patients at least 18 years of age, who have stable angina or acute coronary syndrome (unstable angina or non-ST-segment elevation myocardial infarction) of documented ischemia due to a significant lesion in a native coronary artery, will be included in this study. Patients are eligible for inclusion if the native coronary lesion is >50% stenosed by visual estimation on coronary angiogram with reference diameter between 2.5 and 4.0 mm and lesion length <28 mm. Patients presenting with the following will be excluded from the study: ST-segment elevation myocardial infarction, intended bifurcation stenting, cardiogenic shock, chronic total occlusions, and pregnancy. All inclusion and exclusion criteria are summarized in Appendix A and Appendix B, respectively.

If all of the inclusion criteria are met and none of the exclusion criteria apply, the patients will be asked for written informed consent, as required by the institutional review board in accordance with the Declaration of Helsinki.

### Randomization and interventions

After the patients are enrolled in the present study, patient randomization will be done via a random number table, which will be independently managed at the Seoul National University Bundang Hospital Cardiovascular Research Center.

### Index percutaneous coronary intervention

After random assignment to DEBs first with BMS or DES, the index PCI procedure must be carried out in all patients within 7 days. All patients will receive 300 mg aspirin and a loading dose of 300 to 600 mg clopidogrel before the procedure, unless the patient has been taking these medications for at least for 1 week prior to the procedure. Heparin will be administered intravenously in boluses to maintain an activated clotting time >250 seconds during the procedure. Administration of glycoprotein IIb/IIIa inhibitors is left to the physician’s discretion. PCI will be performed according to current international guidelines. The goals of the procedure are to achieve optimal angiographic efficacy of PCI in selected target lesion sites while minimizing the risk of procedure-related complications. A full range of commercially available guiding catheters, balloon catheters, and guidewires will be readily available. PCI may be performed by the femoral approach. After obtaining coronary angiograms, patients will undergo sequential predilatation with regular balloon of target lesion, after which they are randomly assigned to the DEB first followed by BMS group or the DES group.

In the DEB first followed by BMS group, a gentle balloon deployment will be attempted; otherwise, in case of resistance, additional predilatation with a regular balloon will also be recommended. The length of the DEB will be longer than the BMS to avoid implantation of the BMS on the lesion that is not covered by the DEB. If DEB delivery to the lesion fails due to calcification or severe tortuosity, DES will be inserted into the lesions.

### Quantitative coronary angiography

The coronary angiograms recorded at baseline and at the 9-month follow-up will be analyzed using an automated edge detection system (CASS 5.7.1; Pie Medical Imaging Systems, Maastrict, the Netherlands). In each patient, quantitative coronary angiography measures within the stent and the analysis segment (including the stented region and 5 mm edge regions) will be analyzed and reported separately. In-segment late loss will be defined as the difference between the minimum lumen diameter post procedure and at 9 months. Binary restenosis will be defined as >50% diameter stenosis.

### Intravascular ultrasound

Intravascular ultrasound (IVUS) will be recommended to all patients enrolled in the present study, which will be performed before DEB or DES deployment to assess an adequate size of the balloon or stent, post index procedure and at the 9-month follow-up. IVUS imaging will be performed with a 20 MHz 2.9 F, phased-array IVUS catheter (Eagle Eye; Volcano Therapeutics, Rancho Cordova, CA, USA) after fist administering nitroglycerin (200 mg). IVUS will be performed after optimal results of the index procedure, which be decided by the operating physician based on angiographic results. If the IVUS indicates that the procedural results are not optimal, whether to perform further post-dilatation or bailout stenting will be left to the operator’s discretion. If post-dilatation or bailout stenting is performed, IVUS should be repeated after final post-dilatation or stenting. At the 9-month follow-up, an IVUS image will be obtained after angiography.

### Post-percutaneous coronary intervention medication

All patients included in this trial will be treated according to the current American College of Cardiology/American Heart Association guidelines regarding post-stenting management, which specify treatment with at least 100 mg aspirin daily and 75 mg clopidogrel daily for at least 12 months after PCI.

### Follow-up

Clinical follow-up will be at specified time points (Table 1). Follow-up sessions should be office visits, but telephone contact will be allowed. Data collected during all follow-up visits will include angina class and major adverse ischemic, neurologic and bleeding events, including rehospitalization, recatheterization and adverse events/serious adverse events. Original source documents must be submitted for any clinical events (death, reinfarction, revascularization, stroke, or any other serious adverse effect within the 9-month follow-up). If the patient is readmitted to a nonstudy hospital, all possible efforts should be made to obtain original source documents from that hospital. For all reinfarctions, electrocardiograms and cardiac enzymes (creatine phosphokinase, creatine kinase-MB, Troponin) must be obtained and recorded.

Routine angiographic follow-up at 9 months (−3 months/+3 months) will be recommended in this study. Any earlier angiogram >30 days showing restenosis or thrombosis (diameter stenosis >50%) will qualify as an endpoint angiogram. If an angiogram is performed between 1 and 4 months and restenoses are not present in any study lesion, the requirement for the 9-month angiogram has not been met and thus the 9-month angiographic follow-up must still be performed. Even when there is unexpected angiography between 4 and 6 months and restenosis or thrombosis are not present in any study lesion, the 9-month angiographic follow-up must still be performed. However, unscheduled angiograms >6 months after procedure will be considered as the 9-month follow-up angiogram in the final analysis. Copies of angiograms must be submitted to the angiographic core laboratory of the Seoul National University Bundang Hospital Cardiovascular Research Center. Angiograms to be received by the core laboratory include: the baseline angiogram from all randomized patients; and the 9-month follow-up angiograms.

### Statistical considerations

#### Sample size calculation

The study objective is to determine whether the PCI with DEB first strategy will be inferior to the currently accepted standard of DES for treatment of *de novo* lesions in patients with stenosis in native coronary arteries. Based on angiographic outcomes reported for the PEPCAD III trial [16] and the Endeavor Resolute first-in-man trial [18], we postulated the following in-stent late loss values: DEB first strategy, 0.41 ± 0.51 mm; and DES (zotarolimus-eluting stent), 0.12 ± 0.26 mm. To claim the DEB first strategy non-inferior to the zotarolimus-eluting stent, we assumed a non-inferiority margin of 0.1 mm as the acceptable difference – which is <50% of a difference for in-stent late loss between BMS pre-mounted on DEBs and DES in the PEPCAD III trial, a two-sided alpha-level of 0.05, a statistical power of 80%, and an estimated attrition rate of 20% (for 9-month clinical follow-up). Accordingly we would need a total of 180 patients: 90 patients in the DEB first strategy arm and 90 patients in the zotarolimus-eluting stent arm. This number of patients would also have 85% power to detect superiority with a late luminal loss difference of 0.2 mm between the groups at a two-sided alpha-level of 0.05.

### Statistical analyses

All primary and secondary endpoints will be analyzed both on an intention-to-treat basis (all patients analyzed as part of their assigned treatment group) and on a per-protocol basis (patients analyzed as part of their assigned treatment group only if they actually received their assigned treatment).

Multivariate predictors of all primary and secondary endpoints will be determined using multivariate regression models. Forward stepwise selection algorithms will be used to select independent predictors. Baseline characteristics of study patients will be summarized in terms of frequencies and percentages for categorical variables and by means with standard deviations for continuous variables. Categorical variables will be compared by Fisher’s exact test. Continuous variables will be compared by the two-sample *t* test. *P* = 0.05 will be established as the level of statistical significance for all tests. All time-to-event outcomes will be summarized using Kaplan–Meier survival estimates and compared between treatment groups using log-rank tests. Major subgroup analyses of the primary and major secondary endpoints will be performed; diabetes mellitus, left main lesions, advanced age (age ≥70), renal dysfunction (calculated creatine clearance ≤60 ml/minute) and multivessel stenting.

### Trial organization

#### Executive Committee

The Executive Committee will be composed of the study chairperson and the principal investigators of the investigating centers. This committee will approve the final trial design and protocol issued to the Data Safety Monitoring Board (DSMB) and the clinical sites. The Executive Committee will also be responsible for reviewing the final results, determining the methods of presentation and publication, and selection of secondary projects and publications by members of the Steering Committee.

### Data Safety Monitoring Board

The DSMB is composed of general and interventional cardiologists and a biostatistician. The DSMB will function in accordance with applicable regulatory guidelines. The board members are independent and will not be participating in the trial. The DSMB committee will review the safety data from this study and make recommendations based on safety analyses of unanticipated device effects, serious adverse events, protocol deviation, device failures, and 30-day follow-up reports. The frequency of the DSMB meetings will be determined prior to study commencement. Additionally, the DSMB may call a meeting at any time if there is reason to suspect that safety is an issue.

All cumulative safety data will be reported to the DSMB and reviewed on an ongoing basis throughout enrollment and follow-up periods to ensure patient safety. Every effort will be made to allow the DSMB to conduct an unbiased review of patient safety information. All DSMB reports will be made available to the appropriate agencies upon request but will otherwise remain strictly confidential.

### Clinical Events Adjudication Committee

The Clinical Events Adjudication Committee (CEAC) is comprised of interventional and non-interventional cardiologists who are not participants in the study. The CEAC is charged with the development of specific criteria used for the categorization of clinical events and clinical endpoints in the study that are based on protocol. At the onset of the trial, the CEAC will establish explicit rules outlining the minimum amount of data required, and the algorithm followed in order to classify a clinical event. All members of the CEAC will be blinded to the primary results of the trial.

The CEAC will meet regularly to review and adjudicate all clinical events in which the required minimum data are available. The committee will also review and rule on all deaths that occur throughout the trial.

### Ethical approval

This study has been approved by institutional review board of Seoul National University Bundang Hospital.

## Discussion

### Outcomes of percutaneous coronary intervention with drug-eluting stents

The incidence of in-stent restenosis has been reported as 5 to 35% after BMS implantation [1,2,19]. Although the rates of both clinical and angiographic restenosis are significantly reduced with the DES compared with the BMS, the rate of in-stent restenosis is as high as 19% after implantation of DES in patients at moderate risk [19]. In addition, rapid acceptance of the DES in real-world practice has resulted in the common placement of DES in clinical settings, and these off-label patients have higher rates of adverse events including repeat revascularization [20]. This increase probably contributes to the persistent 5 to 7% incidence of clinical restenosis reported in contemporary PCI registries [21]. In a recent study of long-term DES efficacy, delayed neointimal hyperplasia after silorimus-eluting stent implantation was demonstrated [3,4]. In this study, in-stent neoatherosclerosis was considered an important mechanism of DES failure, especially late after DES implantation [8]. Furthermore, concerns have been raised that DES, although effective, require long durations of antiplatelet therapy to avoid late thrombotic complications [11]. Patients suffering from poor DES outcomes therefore necessitate a search for new methods to prevent target vessel revascularization.

### Advantage and concept of the drug-eluting balloon

Restenosis due to neointimal hyperplasia is a slow process, suggesting that prolonged local drug administration would be needed to be beneficial. Stent-based local drug delivery provides sustained drug release using special release technologies such as polymer coatings. Sustained drug release seems to be essential for stent-based local drug release due to the inhomogeneous drug distribution from DES to the arterial wall. Consequently, relatively high drug concentrations on the stent struts including a controlled and sustained release are required for stent-based local drug delivery, which consequently results in delayed and incomplete endothelialization of the stent struts [22].

A recent study indicated that even brief contact between vascular smooth muscle cells and lipophilic taxane compounds could inhibit vascular smooth muscle cell proliferation for an extended period of time [23-25]. Nonstent-based local drug delivery, particularly a DEB, could homogeneously administer the antiproliferative drug to the vessel wall. The drug concentration at the vessel wall would be the highest at the time of injury when the neointimal process is the most vigorous [10]. In addition, the DEB is a regular angioplasty balloon requiring no special handling. The DEB thus represents a novel option for the treatment of coronary and peripheral arteries and for high-risk restenotic lesions such as small vessels, bifurcations or in-stent restenotic lesions.

Results from a porcine animal model study showed that the drug coated on the balloons of percutaneous transluminal coronary angioplasty or percutaneous transluminal catheters inhibited neointimal hyperplasia [12]. In this study, the most pronounced reduction of neointimal formation was seen with paclitaxel-coated balloon catheters.

### Previous reports of the drug-eluting balloon in percutaneous coronary intervention

Paccocath ISR I was a first-in-man study that investigated the use of paclitaxel-coated balloon catheters for the treatment of coronary in-stent restenosis after BMS implantation. The patients who were treated with the coated balloon had significantly better angiographic results and concomitant improvement in 12-month clinical outcomes compared with patients treated with an uncoated balloon [9]. In the treatment of in-stent restenosis after DES, paclitaxel-coated balloon angioplasty was superior to balloon angioplasty alone for the treatment of drug-eluting stent restenosis [26].

The PEPCAD I trial was the first trial using a drug-eluting balloon in *de novo* coronary narrowing in a high-risk patient population. The PEPCAD I 30-day follow-up results confirmed the safety of the paclitaxel-coated balloon in patients with *de novo* lesions in small coronary arteries [22]. Unverdorben and colleagues reported that treatment of coronary stenosis with the paclitaxel-coated balloon was well tolerated and may offer an alternative to the implantation of a drug-eluting stent for treatment of small coronary vessels [14]. In bifurcation lesion, percutaneous treatment with a DEB showed DES-like results in the main branch and side branch on follow-up angiography at 9 months [15]. However, the Drug-Eluting Balloon in Bifurcations Trial (DEBIUT) study revealed that pretreatment of both the main and side branches with the DEB failed to show angiographic and clinical superiority over conventional BMS, using a provisional T-stenting technique [27]. However, the DEBIUT study was performed using the DIOR-I (Eurocor GMbH, Bonn, Germany) DEB, which is different from other published studies performed with the Sequent please DEB that demonstrated a beneficial effect. The author of the DEBIUT study suggested that the DIOR-I DES may have been insufficient to provide benefits, in terms of late luminal loss, comparable with those observed in the DES arm [27,28].

### Rationale of the drug-eluting balloon with bare metal stents

With the use of a paclitaxel-coated balloon catheter, administration of the antiproliferative drug is homogeneously distributed to the vessel wall in high concentration. Hence, the combination of a paclitaxel-coated balloon plus BMS addresses both issues: reduction of neointimal proliferation due to homogeneous administration of paclitaxel to the vessel wall with high concentration; and reduction in the risk of stent thrombosis by facilitating more rapid endothelialization due to using BMS rather than DES [28]. The DEB with BMS protocol also allows the length of the paclitaxel-coated balloon to be longer than the stented segment. This may be favorable since about one-third of restenosis after DES implantation occurs proximal or distal to the stent margin [19,28].

The PEPCAD III trial compared BMS mounted on a DEB with the sirolimus-eluting stent to treat *de novo* stenosis in native coronary arteries. However, the study demonstrated that the BMS pre-mounted on DEB strategy did not meet the non-inferiority criteria versus the sirolimus-eluting stent [16]. In PEPCAD III, since the BMS pre-mounted on the DEB was implanted in the *de novo* lesion, the stent strut may have prevented drugs from being appropriately delivered and uniformly coating the diseased vessel wall. Consequently, the BMS mounted on DEB strategy of PEPCAD III may have resulted in the different efficacy and mechanism of action compared with previous DEB studies. This strategy might diminish the efficacy of the DEB shown in previous DEB studies that did not demonstrate non-inferiority compared with the DES.

We therefore designed a clinical study with a different protocol, in which the DEB is deployed first followed by BMS implantation in comparison with drug-eluting stent implantation. From the protocol results, we expect to demonstrate the combined efficacy of DEB and BMS in the treatment of *de novo* coronary lesions.

In conclusion, this study is the first randomized controlled trial of a DEB first followed by BMS implantation for the treatment of *de novo* coronary lesions. The study may also shed light on whether the efficacy and safety of a DEB followed by BMS is non-inferior to DES in patients with *de novo* coronary lesion.

## Trial status

The trial is currently in the recruitment phase.

## Appendix A. Inclusion criteria

• Patients with stable or acute coronary syndrome (unstable angina or non-ST-segment elevation myocardial infarction) or documented ischemia due to a significant lesion in a native coronary artery.

• Patients eligible for coronary revascularization by means of percutaneous coronary intervention.

• Patients must be ≥18 years of age.

• Women of childbearing potential may not be pregnant nor have the desire to become pregnant during the first year following the study procedure. Hence, patients will be advised to use an adequate birth control method up to and including the 9-month follow-up.

• Patients who are mentally and linguistically able to understand the aim of the study and to show sufficient compliance in following the study protocol.

• Patients must agree to undergo the 9-month angiographic follow-up.

• Patient is able to verbally acknowledge an understanding of the associated risks, benefits, and treatment alternatives to the therapeutic options of this trial; for example, balloon angioplasty by means of the paclitaxel-eluting PTCA-balloon catheter in combination with the Coroflex Blue stent or the Resolute integrity stent. The patients, by providing informed consent, agree to these risks and benefits as stated in the patient informed consent document.

• Significant stenoses in native coronary arteries with nominal stent diameters between ≥2.5 mm and ≤4.0 mm and ≤28 mm in length.

## Appendix B. Exclusion criteria

• Unprotected left main lesion.

• In-stent restenosis.

• Intended bifurcational stenting.

• Patients requiring chronic anticoagulation.

• ST-segment elevation myocardial infarction.

• Cardiogenic shock.

• Chronic total occlusions.

• Pregnancy.

• Patients with standalone balloon angioplasty, or stent deployment 6 months prior to enrolment into this study.

• History of cerebrovascular accident or myocardial infarction within 1 year.

## Abbreviations

CEAC: Clinical Events Adjudication Committee; DEB: drug-eluting balloon; DEBIUT: Drug-Eluting Balloon in Bifurcations Trial; DES: drug-eluting stents; DSMB: Data Safety Monitoring Board; IVUS: intravascular ultrasound; PCI: percutaneous coronary intervention; BMS: bare metal stents; PEPCAD: Paclitaxel Eluting PTCA Balloon in Coronary Artery Disease.

## Competing interests

The authors declare that they have no competing interests.

## Authors’ contributions

C-HY and I-HC participated in the conception and design of the study. C-HY and S-DP contributed equally to the preparation of this manuscript. I-YO, J-WS, Y-SC, T-JY, and D-JC will enroll patients and collect clinical and IVUS data. S-DP and C-HY will analyze IVUS and coronary angiographic images in a core laboratory. I-HC is the principal investigator and initiator of the study, designed the study and supervised and participated in writing the manuscript. All authors read and approved the final manuscript.
