# Erratum to: Comparison of a drug-eluting balloon first and then bare metal stent with a drug-eluting stent for treatment of de novo lesions: study protocol of a randomized controlled trial

**DOI:** 10.1186/s13063-017-1793-y

**Published:** 2017-05-23

**Authors:** Sang-Don Park, Chang-Hwan Yoon, Il-Young Oh, Jung-Won Suh, Young-Suk Cho, Tae-Jin Youn, Dong-Ju Choi, In-Ho Chae

**Affiliations:** Division of Cardiology, Department of Internal Medicine, Seoul National University Bundang Hospital, 82 Gumi-ro, 173 Bein-gil, Bundang-gu, Seongnam-si, Gyeonggi-do 463-707 Republic of Korea

Unfortunately, the original version of this article [1] contained an error. The correct paragraph can be found below:

The calculation of number of patients was not correct. It was wrote the non-inferiority margin as 0.1 mm. But it was 0.19 mm in an original document. Non-inferiority margin in previous coronary intervention studies has been set between 0.1 ~ 0.2 mm. The 0.19 mm is not irrelevant in view of the current trial standard.
